# Supplementary material for: Environment-specificity and universality of the microbial growth law
Source: Commun Biol. 2022 Aug 31;5:891. doi: 10.1038/s42003-022-03815-w (PMC9433384; doi:10.1038/s42003-022-03815-w)
Supplement: Supplementary file 2 — Supplementary Information [file 42003_2022_3815_MOESM2_ESM.pdf]

Supplementary Information

Qirun Wang[1] and Jie Lin[1,2]
[1] *Center for Quantitative Biology, Peking University, Beijing, China*
[2] *Peking-Tsinghua Center for Life Sciences, Peking University, Beijing, China*

Table S1: A summary of the parameters used in the numerical simulations not mentioned in the main text.

Parameters	Meaning	Values
N	number of genes	4.00×10^3
m_R	molecular mass of the ribosome	1.40×10^6 Da
ϕ_0	the mass fraction of inactive ribosomes	8.00×10^{-2}
$\langle k \rangle$	the average mass of translated non-ribosomal proteins per unit time	4.80×10^4 Da/min
CV_k	The coefficient of variation for the distribution of k_i	1.97
k_R	the mass of translated ribosomal protein per unit time	2.07×10^4 Da/min
$\langle \alpha \rangle$	the average degradation rate of non-ribosomal proteins	1.10×10^{-3} 1/min
CV_α	The coefficient of variation for the distribution of α_i	1.76
α_R	degradation rate of the ribosomal protein	4.83×10^{-4} 1/min
CV_χ	The coefficient of variation for the distribution of χ_i	4.5-5.5

Table S2: A summary of the Pearson correlations between the predicted ϕ_i and experimental ϕ_i. We also show the correlation between the experimental χ_i and ϕ_i.

Data	WT+2%glu	WT+2%gly	*natA*Δ+2%glu	*natA*Δ+2%gly
$\rho_{\phi,\chi}$	0.82	0.75	0.83	0.70
$\rho_{\phi_{pre},\phi}$	0.68	0.31	0.79	0.26
$\rho_{\phi_{pre},\phi}$ with $\alpha_i = 0$	0.69	0.23	0.80	0.22
$\rho_{\phi_{pre},\phi}$ with $k_i = \langle k \rangle$	0.82	0.76	0.82	0.78
$\rho_{\phi_{pre},\phi}$ with $\alpha_i = 0$ and $k_i = \langle k \rangle$	0.82	0.75	0.83	0.70

Table S3: A summary of the variables used in this work.

Variables	Meaning
N	number of genes
M_i	mass of protein i
M	total mass of all proteins
k_i	the mass of translated protein i per unit time
k_R	the mass of translated ribosomal protein per unit time
$\langle k \rangle$	the arithmetic average mass of translated protein mass over non-ribosomal proteins per unit time
$\langle k \rangle_\chi$	the $\chi-$weighted average mass of translated non-ribosomal proteins per unit time
χ_i	the fraction of active ribosomes producing protein i in the pool of total active ribosomes
χ_R	the fraction of active ribosomes producing themselves in the pool of total active ribosomes
$\widetilde{\chi}_i$	the fraction of active ribosomes producing protein i in the pool of active ribosomes translating non-ribosomal proteins
R	total number of ribosomes
R_0	total number of inactive ribosomes
α_i	degradation rate of protein i
α_R	degradation rate of the ribosomal protein
$\langle \alpha \rangle$	the arithmetic average degradation rate over non-ribosomal proteins
$\langle \alpha \rangle_\phi$	the $\phi-$weighted average degradation rate over non-ribosomal proteins
ϕ_i	the mass fraction of protein i
ϕ_R	the mass fraction of ribosomes
ϕ_0	the mass fraction of inactive ribosomes
$\widetilde{\phi}_i$	the mass fraction of non-ribosomal protein i in the pool of all non-ribosomal proteins
m_R	molecular mass of ribosome
μ	the growth rate
$I_{\chi,k}$	the metric quantifying the correlation between the ribosome allocations and the translation speeds of non-ribosomal proteins
$I_{\phi,\alpha}$	the metric quantifying the correlation between the mass fractions and the degradation rates of non-ribosomal proteins

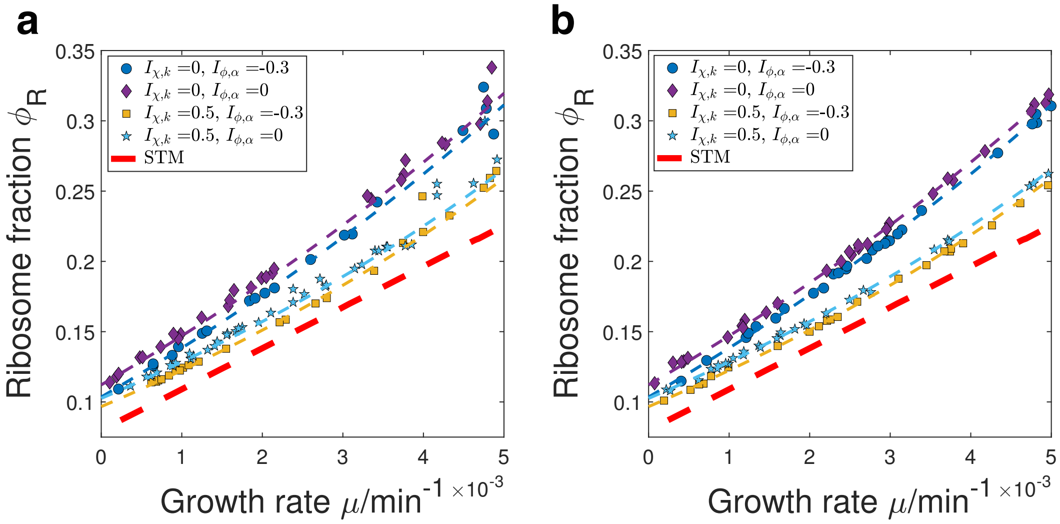

Figure S1: Simulations including random elongation speeds and random allocation fractions of ribosomes. Simulation details are the same as the main text, except that we add noises to the elongation speed k_i and the allocation fraction of ribosomes χ_i, respectively. (a) Simulations including random elongation speeds. We add a normal distributed noise $\xi_k \sim N(0, (\frac{k_i}{10})^2)$ to the elongation speed of each protein. (b) Simulations including random allocations of ribosomes. We add a normal distributed noise $\xi_\chi \sim N(0, (\frac{\chi_i}{10})^2)$ to the allocation fraction χ_i and then normalize them so that the sum of χ_i equals 1.

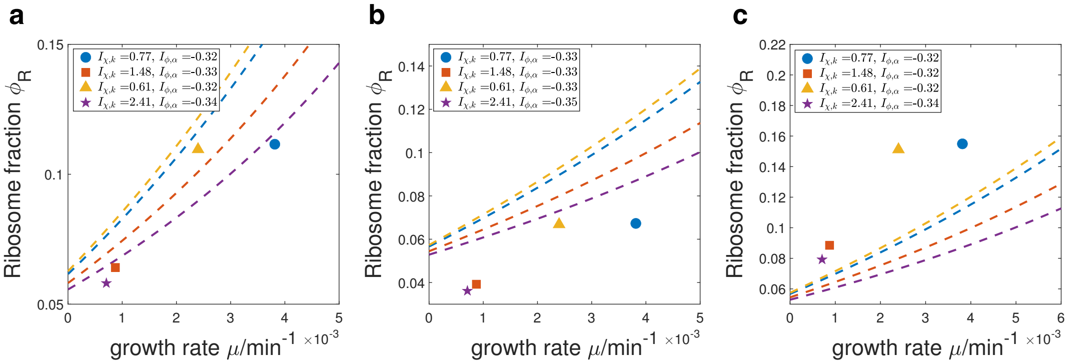

Figure S2: Experimental tests of the theoretical predictions of ϕ_R with different data processing procedures. $\phi_0 = 0.048$ are used in all the cases. (a) The case in which the actual ribosomal mass $m_R = 1.40 \times 10^6$ Da is used in the predictions. (b) The case in which the experimental ϕ_i is not calibrated. (c) The case in which the experimental ϕ_i is calibrated with L^{-1}.

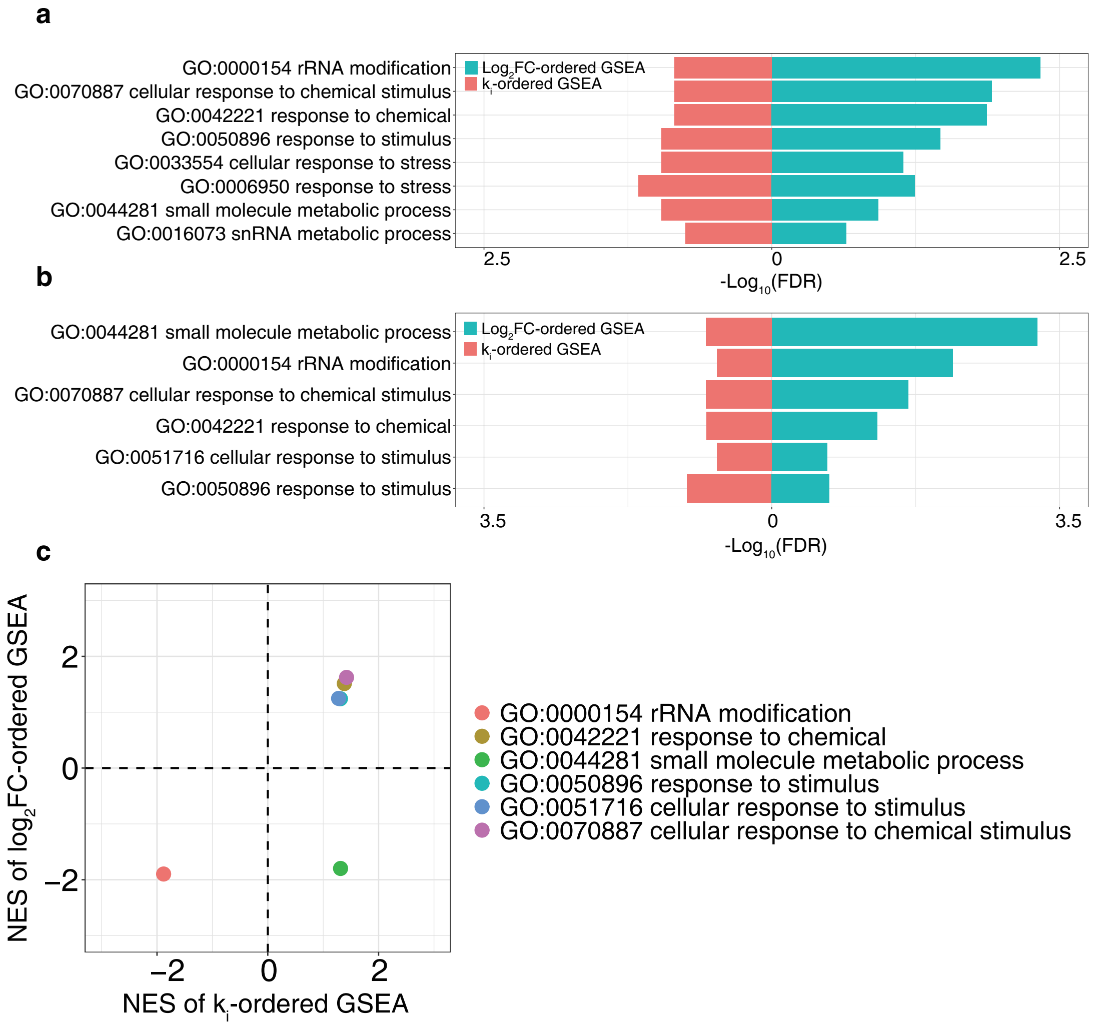

Figure S3: GSEA results of WT and $natA\Delta$ cells. (a) The enriched gene sets for WT cells with their false discovery rate (FDR) q values of the single-sided permutation test. The higher the $-\log_{10}(\text{FDR})$ value is, the more likely a gene set is enriched. (b) The enriched gene sets for $natA\Delta$ cells with their false discovery rate (FDR) q values. (c) The normalized enrichment score (NES) of GSEA of enriched gene sets for $natA\Delta$ cells. A positive NES of k_i-ordered GSEA means that the genes in the corresponding gene set are enriched in the regime of higher k_i. A positive NES of $\log_2$FC-ordered GSEA means that the genes in the corresponding gene set are enriched in the regime of increasing χ_i when the nutrient changes from glucose to glycerol.
