# Supplementary material for: Environment-specificity and universality of the microbial growth law
Source: Commun Biol. 2022 Aug 31;5:891. doi: 10.1038/s42003-022-03815-w (PMC9433384; doi:10.1038/s42003-022-03815-w)
Supplement: Supplementary file 3 — Description of Additional Supplementary Files [file 42003_2022_3815_MOESM3_ESM.pdf]

Description of Additional Supplementary Files

File name: Supplementary Data 1
Description: Ribosome elongation speeds k_i of budding yeast.

File name: Supplementary Data 2
Description: Protein mass fraction φ_i of budding yeast calibrated with $L^{-0.57}$.

File name: Supplementary Data 3
Description: : Ribosome allocation χ_i of budding yeast.

File name: Supplementary Data 4
Description: : Growth rates μ in different conditions of budding yeast.
